# Repair of Left Ventricular Pseudoaneurysm Due to Systemic Lupus Erythematosus

**DOI:** 10.21470/1678-9741-2024-0038

**Published:** 2025-02-11

**Authors:** Mehmet Işık, Niyazi Görmüş

**Affiliations:** 1 Department of Cardiovascular Surgery, Faculty of Medicine, Necmettin Erbakan University, Konya, Turkey.

**Keywords:** Left Ventricular Pseudo-Aneurysm, Systemic Lupus Erythematosus, Ventriculoplasty

## Abstract

Systemic lupus erythematosus is a clinically heterogeneous autoimmune disease that frequently affects young women. The risk of cardiovascular events is higher in patients with this disease than in the general population. In this study, we report a patient who developed a left ventricular pseudoaneurysm with no etiological factor other than systemic lupus erythematosus. Dor ventriculoplasty and mitral ring replacement were performed as surgical treatment. The case is shared because of the rarity of left ventricular pseudoaneurysm due to systemic lupus erythematosus and successful pseudoaneurysm repair.

## INTRODUCTION

Systemic lupus erythematosus (SLE) is a clinically heterogeneous autoimmune disease that frequently affects young women. The risk of cardiovascular events is twice as high in patients with SLE compared to the general population^[1,2]^.

The most common causes of left ventricular pseudoaneurysm (LVP) include myocardial infarction, cardiac surgery, cardiac tumor, trauma, and infective endocarditis^[3]^. Ventricular aneurysm after myocardial infarction has been reported with a rate of 22%, while pseudoaneurysm has been reported to be < 0.5%^[4,5]^.

In this study, we present a patient who developed LVP in which no etiological factor other than SLE could be found. Dor ventriculoplasty and mitral ring replacement were performed as surgical treatment. The case is shared because of the rarity of LVP due to SLE and successful pseudoaneurysm repair.

## CASE PRESENTATION

**Fig. 1:**
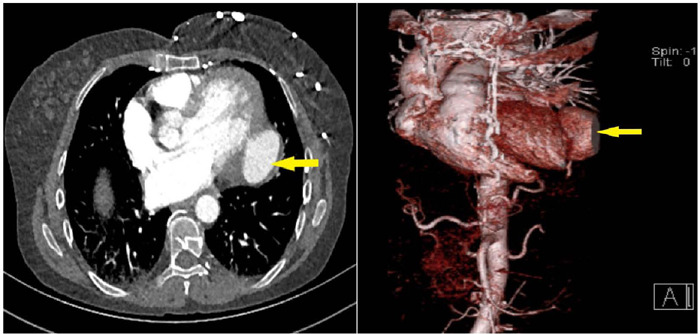
Computed tomography angiography image of left ventricular aneurysm (arrows).

**Fig. 2:**
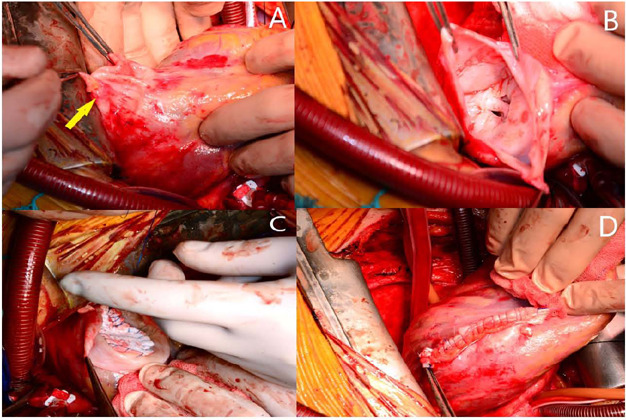
A) to D) Surgical images of the left ventricular aneurysm (arrow) repair.

A 57-year-old woman complained of dyspnea on exertion for one month. She had a history of SLE and systemic sclerosis for eight years and hypertension for five years. Despite detailed questioning, there was no history of previous trauma or any cardiac intervention. Hydroxychloroquine sulfate, methyl prednisolone, and calcium channel blocker were the medications she was taking continuously. Laboratory tests showed hemoglobin 10.7 g/dl; leukocyte and platelet counts were normal. Creatinine was 0.98 mg/dl, urea was 26.7 mg/dl, and albumin was 4.3 g/dl. Transthoracic echocardiography showed an ejection fraction of 40%, moderate mitral regurgitation, pulmonary artery pressure of 35 mmHg, fibrocalcific aortic valve cusps with a gradient of 20/11 mmHg, and a 5 × 3.5 cm pseudoaneurysm in the posterolateral wall of the left ventricle. Thoracoabdominal computed tomography angiography showed a narrow-necked, approximately 5 × 3 cm pseudoaneurysm on the posterolateral wall of the left ventricle (Figure 1). On cardiac magnetic resonance imaging, a 5-cm diameter thin-necked aneurysm was observed adjacent to the left ventricle. As a result of the Cardiology - Cardiovascular Surgery council, the patient was decided to undergo surgery. Preoperative coronary angiography was performed, and no pathology was observed except slow flow in the right coronary artery. Under general anesthesia, right atriotomy and septostomy were performed after sternotomy. A No. 30 Medtronic brand ring was placed in the mitral valve position. The septum and right atriotomy were closed. Then, the aneurysm tissue in the posterior lateral part of the left ventricle was excised. A Teflon cardiac patch (approximately 3 × 2 cm) was placed in this area. And the aneurysm tissue was closed linearly on the patch with the help of Teflon felt (Dor ventriculoplasty) (Figure 2). The patient was extubated at the fifth postoperative hour and discharged on the sixth postoperative day. At one-month follow-up echocardiography, ejection fraction was 45%, there was mild mitral regurgitation, pulmonary artery pressure was 30 mmHg, and aortic valve cusps were fibrocalcific with a gradient of 20/10 mmHg.

## DISCUSSION

The reasons for the frequent coexistence of rheumatic diseases and cardiovascular diseases include common inflammatory mediators, post-translational modifications of peptides/proteins and subsequent immune responses, changes in the composition and function of lipoproteins, increased oxidative stress, and endothelial dysfunction^[6]^. It has been reported that more left ventricular strain is observed in rheumatoid arthritis patients and that this condition is related to the activity of the disease^[6]^. Similarly, it is stated that higher rheumatologic activity increases the left ventricular global length^[7]^.

A case with normal coronary arteries and LVP secondary to SLE was identified in the literature^[8]^. It has been reported that pseudoaneurysm may be a result of myocardial necrosis due to small vessel vasculitis, most likely secondary to SLE activity^[8]^. In another study, cases of myocardial rupture secondary to SLE were reported^[1,9]^. In this study, it is stated that since the coronary angiography was normal, the possible etiological cause may be SLE-related microcirculation thrombosis or an embolic condition resulting in rupture. Although less likely, it has been reported that SLE-related myocarditis may cause heart rupture^[9]^. Despite the fact that the etiology of pseudoaneurysm was not clear in our case, it was thought to be due to SLE because of the absence of a history of trauma, cardiac intervention, or surgery, normal coronary angiography, and exclusion of antiphospholipid syndrome.

Ventricular aneur ysms or pseudoaneurysms are important because they can cause embolism, rupture, and arrhythmia. Although there is no consensus on the treatment of LVP, it has been reported that LVPs occurring within the first three months after acute myocardial infarction require emergency surgery, especially if they are > 3 cm in diameter^[4]^. In chronic cases, there is no consensus because the risk of rupture decreases as the left ventricular cavity stabilizes and because of the high mortality of surgical treatment. Perioperative mortality has been reported to be around 10-20%^[4]^. In this case, the duration of the patient's pseudoaneurysm was unknown. However, the fact that his complaints had been present for one month suggests an acute-subacute stage. Surgery was decided because of the large LVP diameter (5 × 3.5 cm) and moderate mitral regurgitation.

## CONCLUSION

In conclusion, LVP occurs mostly after myocardial infarction, but in rare cases it can also present with atypical etiologies such as SLE. Although surgical treatment is a concern because of the high mortality rate, successful ventriculoplasty can avoid destructive complications.
